# Cold‐Induced Symptoms in a Young Female: A Perioperative Case of Cold Urticaria for Gynecologic Surgery—A Case Report

**DOI:** 10.1155/cria/5784668

**Published:** 2026-06-15

**Authors:** Keevan Singh, Lance De Barry, Vishal Bahall

**Affiliations:** ^1^ Department of Anaesthesia and Intensive Care, San Fernando General Hospital, South-West Regional Health Authority, San Fernando, Trinidad and Tobago, health.gov.tt; ^2^ Department of Obstetrics and Gynaecology, San Fernando General Hospital, South-West Regional Health Authority, San Fernando, Trinidad and Tobago, health.gov.tt

**Keywords:** anaphylaxis, case report, cold, perioperative, urticaria

## Abstract

**Background:**

Cold urticaria is a rare subtype of chronic inducible urticaria that is associated with the development of localized or systemic symptoms following exposure to cold stimuli. Although considered benign, it may be associated with severe reactions, including anaphylactoid reactions. This can occur upon direct or indirect exposure to cold temperatures, liquids, and objects. The perioperative environment contains multiple potential cold triggers which can cause generalized hypersensitivity reactions in these patients. Little is known of the perioperative course and management of these patients, and there are few reported cases. Treatment of cold urticaria involves the use of antihistamine medication and avoidance of cold triggers.

**Case Presentation:**

We describe the case of a young female with undiagnosed cold urticaria that was detected shortly before surgery. A detailed history revealed recurrent cold‐induced symptoms, including a prior episode suggestive of airway compromise. A presumptive diagnosis was made based on clinical history and later confirmed with a cold stimulation test. Perioperative management was modified to minimize cold exposure and include prophylactic antihistamine and corticosteroid therapy. Despite these measures, localized urticarial changes occurred intraoperatively following exposure to cold antiseptic solution on the abdomen without progression to systemic manifestations. The patient remained hemodynamically stable throughout and had an uneventful postoperative recovery.

**Conclusion:**

This case highlights the importance of early recognition of cold urticaria and the implementation of targeted perioperative strategies to reduce the risk of adverse events. Currently, there are no standardized perioperative guidelines for cold urticaria, and given that patients may not spontaneously report cold‐induced symptoms, specific screening questions during preoperative assessment should be considered to improve detection and optimize perioperative safety.

## 1. Introduction

Intraoperative anaphylaxis is a potentially fatal condition during surgery and anesthesia. A recent report in the United Kingdom revealed an incidence of 1 in 10,000 anesthetics with 10 deaths and 40 cardiac arrests reported over a 1‐year period [1]. Common agents used during anesthesia, such as antibiotics and neuromuscular blocking agents, were found to be the most common precipitants [1].

Urticaria is a common allergic condition with a prevalence of up to 11% in the general population and is generally benign [2]. Cold urticaria (ColdU) is a subtype of urticaria with a much lower prevalence of 0.05% but can be associated with life‐threatening anaphylactic reactions on exposure to cold [3]. The diagnosis of ColdU is usually made using patient history and a cold simulation test using ice cubes applied to the forearm [4]. ColdU is of particular relevance in the intraoperative environment, where exposure to cold temperatures and fluids may be unavoidable and thus trigger anaphylaxis.

ColdU, although rarely mentioned in the perioperative literature, has been associated with severe hypotension, angioedema, and hypoxemia intraoperatively [5, 6]. In these cases, the diagnosis was usually made following the episode of anaphylaxis and on further questioning of the patient or relative. Once identified early, effective prophylaxis and preventative measures can be taken, which can allow surgery to safely proceed [7]. Given the fact that urticaria may often be dismissed by physicians, patients may not be forthcoming with symptoms, leading to a delay in perioperative diagnosis with potentially serious complications [2].

We describe a case of a young female with an undiagnosed severe ColdU which was detected on preoperative screening. A visible and localized wheal‐and‐flare reaction developed shortly after the induction of anesthesia following the surgical preparation of the abdomen with a cold alcohol solution. There were, however, no anaphylactic‐type features present. This case highlights the challenges associated with this unique condition and the perioperative modifications utilized to achieve an uneventful surgical procedure and a positive patient outcome.

## 2. Case Presentation

A 27‐year‐old female presented to the gynecology clinic with menorrhagia, dysmenorrhea, and symptomatic anemia for six months. She was nulliparous and had future fertility prospects. The patient had no known chronic medical conditions, including asthma or autoimmune disease. She underwent no prior surgeries and was not on any regular prescribed medications or herbal therapies. She denied any known drug allergies.

On clinical examination, a 16‐week‐sized firm uterus was palpated in the pelvis. Speculum examination demonstrated a healthy appearing cervix. Blood investigations revealed a microcytic anemia (hemoglobin 8.74 g/dL and mean corpuscular volume 66.3 fL) and normal renal and liver function tests. Pelvic ultrasonography revealed an enlarged uterus with a 9 × 9 cm posterior wall uterine leiomyoma. The patient was counseled on fertility‐sparing surgery, and she opted for an open myomectomy.

During preoperative teleconsultation with the anesthesiology team one day before surgery, the patient reported that she had a “severe” allergy to cold temperatures. Although initially dismissed, the patient was persistent in her claims. On further questioning, she described recurrent episodes of a raised red rash associated with cold exposure, including a prior episode following immersion in cold seawater at a local beach. During this episode, she developed a generalized urticarial rash accompanied by throat tightness, severe dyspnea, and a sensation of airway narrowing, suggestive of angioedema. She was immediately transferred to a nearby hospital and was told that she experienced a life‐threatening anaphylactic reaction. The patient was unable to confirm what medications were given at that time.

She also reported that ingestion of cold liquids precipitated airway swelling and difficulty breathing. The patient reported that she self‐medicated with oral fexofenadine for symptomatic relief and general prophylaxis before cold exposure. Given the severity and reproducibility of her symptoms, further evaluation was undertaken in consultation with the anesthesiology team during preoperative assessment. At that stage, a presumptive diagnosis of ColdU was made based on clinical history. Her perioperative management plan was modified, and a cold stimulation (ice cube) test was deferred until the postoperative period to avoid provoking a systemic reaction immediately before surgery.

The patient received oral fexofenadine 120 mg and prednisolone 20 mg approximately 12 h prior to surgery, with a repeat dose administered 2 h before induction of anesthesia. The patient had a standard, uneventful induction of anesthesia. Anesthesia was induced using fentanyl 100 μg, propofol 150 mg, and cisatracurium 10 mg. Following intubation, anesthesia was maintained with sevoflurane (2.2%) in an oxygen and air mixture. Prior to skin incision, an ultrasound‐guided transversus abdominal plane (TAP) block was performed bilaterally using 20 mLs of 0.25% bupivacaine per side. A forced air warmer (Bair Hugger) was used intraoperatively to maintain her core body temperature. The forced air warmer was placed on its highest setting (43°C) at the start of the case. Warm intravenous fluids were infused for the case. An infra‐red thermometer was used to measure the axillary temperature throughout the case. Dexamethasone 8 mg IV was given during induction for both its anti‐inflammatory and analgesic effects. Epinephrine was diluted (10 μg/mL) preinduction for emergency intravenous bolus dosing in the event of any life‐threatening anaphylactic reactions.

Standard intraoperative monitoring was employed throughout, including continuous electrocardiography, pulse oximetry, capnography, and repeated noninvasive blood pressure measurements.

**FIGURE 1:**
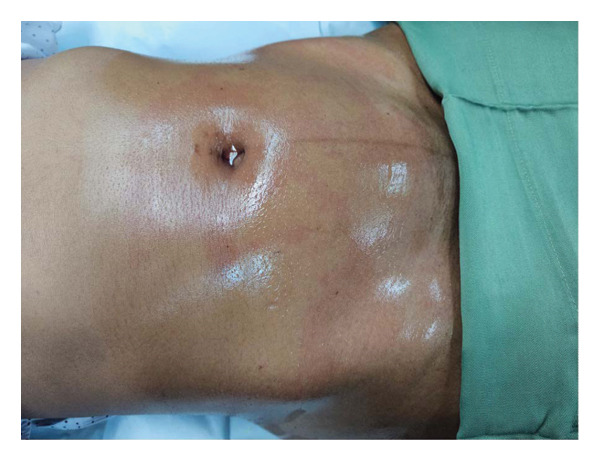
Raised red rash on the abdominal wall that appeared shortly after applying chlorhexidine and alcohol solution.

The skin was prepared with chlorhexidine in alcohol solution for approximately 1 minute. A generalized urticarial rash was observed over the abdomen almost immediately after application of the solution (Figure 1). Despite this, her airway pressures were normal (< 20 cm H_2_O), her mean arterial pressure remained > 65 mmHg, and there were no other signs of anaphylaxis.

Surgical time was approximately 2 h, with four uterine leiomyomas being removed, the largest measuring 9 × 9 cm. Blood loss was minimal, aided by the use of an intraoperative uterine tourniquet. Her axillary temperature was maintained at 36.8°C throughout the case. Prior to extubation, paracetamol 1g IV, parecoxib 40 mg IV, and morphine 5 mg IV were given for analgesia. The patient was successfully extubated at the end of surgery, and active air warming was continued during the postoperative period. No other cutaneous or systemic manifestations were noted. The noted cutaneous reaction on the skin took approximately 4 hours to resolve completely.

A cold stimulation test was performed before discharge. This was achieved by the application of ice cubes on the volar aspect of the patient’s forearm. Approximately 3–5 min after application, a localized wheal‐and‐flare reaction developed, indicating that a positive test was elicited. The patient’s postoperative course was uneventful, and she was discharged on Postoperative day 1.

## 3. Discussion

ColdU presents several unique perioperative challenges, particularly in patients undergoing major surgical procedures. While the exact pathogenesis remains unelucidated, ColdU involves mast cell and basophil degranulation, leading to the localized or systemic release of histamine and other inflammatory mediators that manifest with pruritus, erythema, and localized edema [4, 7]. In a recently completed trial, Bizjak et al. showed an incidence of anaphylactic symptoms (cardiovascular, respiratory symptoms, and gastrointestinal) of 37% in those patients with a positive cold stimulation test [8].

In a review of perioperative allergic conditions, Dewachter et al. reviewed all published cases of patients with ColdU presenting for surgery [7]. Ten cases were reviewed from 1998, with seven of them undergoing major cardiac surgery under cardiopulmonary bypass. These seven cases all received preoperative antihistamine and steroid therapy, with some up to 7 days before surgery. No adverse events were reported in these patients. In two cases in patients undergoing noncardiac surgery, where the diagnosis was not known preoperatively, and no preoperative medication was given, symptoms of intraoperative systemic reactions were noted [5–7]. Triggers for these two patients were thought to be the injection of cold atracurium and the irrigation of the operative site with cool fluids. The diagnosis of ColdU in both these patients was confirmed postoperatively with the ice cube test. This test is performed using melting ice cubes in a thin plastic bag applied to the volar surface of the hand for 5 minutes. A positive test is identified by the formation of a wheal within 10 min of application of the ice cubes [3].

Regarding chronic urticaria, antihistamines are the mainstay of treatment with second‐generation agents regarded as first line [2]. Among second‐generation agents, certain agents may be more efficacious with cetirizine demonstrating higher efficacy than fexofenadine [9]. A higher‐dose antihistamine regimen has also been proven to be very effective in chronic urticaria, with a more than 15% improvement in the rate of response being observed with higher doses [2]. H2‐specific antagonists are generally not recommended, with corticosteroids being recommended for antihistamine‐resistant urticaria, with a preference being for short courses of oral steroids [2].

No specific treatment guidelines exist for the management of chronic or cold‐induced urticaria in the perioperative period. Dewachter et al., in their review, recommend that chronic urticaria should be treated before surgery whenever possible [7]. In their review of the literature, no anaphylactic episodes were recorded in patients who received pretreatment prior to and during surgery, even when hypothermic cardiopulmonary bypass was administered. As with chronic therapy, antihistamines (H1, second generation) are the mainstay, with preoperative doses continued until the day of surgery and preoperative doses added for patients not on antihistamines [7]. Steroids are often combined with antihistamines, with longer preoperative courses being recommended (5–7 days) depending on the nature of cold exposure and severity of symptoms [7]. Higher‐dose antihistamines can also be considered due to their greater efficacy [2].

In patients with severe ColdU, additional therapeutic options may be considered. Omalizumab has shown benefit in selected patients with ColdU who remain symptomatic despite antihistamine therapy [4]. However, its role is primarily in longer‐term disease control rather than urgent perioperative prophylaxis [4]. It was not applicable in the present case, given the late preoperative recognition of the condition and its unavailability in our clinical setting.

In our case, due to the short time between the preoperative assessment and surgery, fexofenadine was combined with oral steroids before surgery and supplemented with intravenous dexamethasone. Despite this, given the appearance of the urticarial rash during cleaning with alcohol‐based solution, it is likely that a higher dose or a longer period of preoperative antihistamine and steroid dose may be required.

**FIGURE 2:**
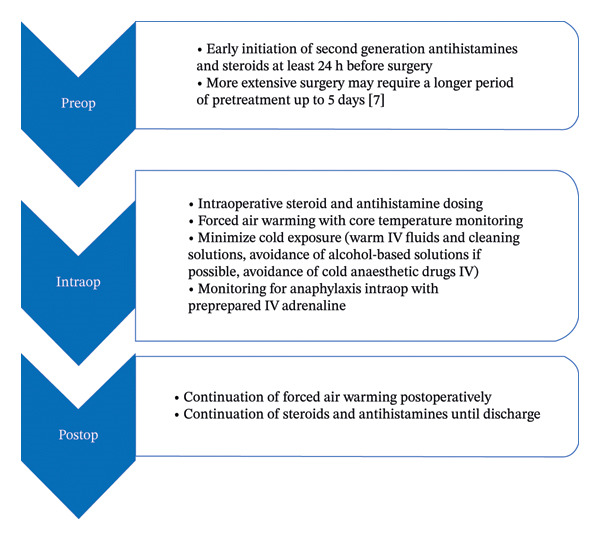
Flowchart showing key management issues in patients with ColdU presenting for surgery.

Fundamental in managing cases of ColdU is minimizing cold exposure [2, 7]. Both general anesthesia and surgical technique expose a patient to significant hypothermia in the intraoperative period. There is an almost linear fall in core temperature during anesthesia due to anesthetic agent‐induced vasodilatation, which can cause as much as a 1.5°C drop in the first hour [10]. Without the use of forced air warming devices, this drop in temperature continues until the vasoconstrictive threshold is reached [10]. This core temperature drop, along with the use of cold irrigation fluids and alcohol‐based solutions, can serve as potent triggers for anaphylactic‐type reactions in patients with ColdU [5–7]. Mitigative measures include active warming, use of chlorhexidine solutions without alcohol, and warm irrigation and intravenous fluids [5–7, 11]. In addition, intravenous injection of cold anesthetic drugs should be avoided [5]. Therefore, due to cold storage requirements, rocuronium may be preferable to atracurium. A flowchart outlining these key management issues is shown in Figure 2.

Lastly, given the rarity of ColdU, many surgeons and anesthesiologists may not be familiar with its presentation and consideration. This was previously alluded to by Sanchez‐Borges, where urticaria is generally considered of little significance by clinicians [2]. This problem can be exacerbated in the perioperative period, where clinicians may have different priorities. This case also underscores the value of structured preoperative interviewing. Patients may not spontaneously report cold‐induced symptoms, especially when previous episodes were self‐limited and never formally diagnosed. Focused questioning regarding rash, swelling, throat tightness, dyspnea, or syncope after exposure to cold air, cold water, cold food, or beverages may improve the recognition of ColdU before surgery and facilitate safer perioperative planning.

Despite the successful outcome in this case, given its limitation of being a single case, generalization may not be possible in other cases with differing severity and surgical complexity.

## 4. Conclusion

ColdU is a rare inducible urticaria that can lead to life‐threatening systemic reactions when patients are exposed to cold stimuli. Due to the potential triggers in the perioperative environment, adequate planning is necessary to minimize morbidity and mortality. Adequate preoperative preparation involves pretreatment with antihistamines, steroids, meticulous temperature control, and reduction of direct contact with cold stimulus.

## Funding

No funding was received for this study.

## Consent

Written informed consent prior to publication was obtained for the use of clinical images and information.

## Conflicts of Interest

The authors declare no conflicts of interest.

## Patient Perspective

The patient was extremely anxious before surgery, fearing cold exposure and its related consequences. She was pleased her symptoms were not ignored and that a comprehensive management plan was put in place with the eventual confirmation of her diagnosis.

## Data Availability

Data sharing is not applicable to this article as no datasets were generated or analyzed during the current study.

## References

[1] Harper N. J. N. , Cook T. M. , Garcez T. et al., Anaesthesia, Surgery, and Life-Threatening Allergic Reactions: Epidemiology and Clinical Features of Perioperative Anaphylaxis in the 6th National Audit Project (NAP6), British Journal of Anaesthesia. (2018) 121, no. 1, 159–171, 10.1016/j.bja.2018.04.014. 29935567

[2] Sánchez-Borges M. , Asero R. , Ansotegui I. J. et al., Diagnosis and Treatment of Urticaria and Angioedema: A Worldwide Perspective, World Allergy Organization Journal. (2012) 5, no. 11, 125–147, 10.1097/WOX.0b013e3182758d6c. 23282382 PMC3651155

[3] Stepaniuk P. , Vostretsova K. , and Kanani A. , Review of Cold-Induced Urticaria Characteristics, Diagnosis and Management in a Western Canadian Allergy Practice, Allergy, Asthma & Clinical Immunology. (2018) 14, no. 1, 10.1186/s13223-018-0310-5. PMC6299577 30574166

[4] Maltseva N. , Borzova E. , Fomina D. et al., Cold Urticaria-What We Know and What We Do Not Know, Allergy. (2021) 76, no. 4, 1077–1094, 10.1111/all.14674. 33249577

[5] Maciag M. C. , Nargozian C. , and Broyles A. D. , Intraoperative Anaphylaxis Secondary to Systemic Cooling in a Pediatric Patient with cold-induced Urticaria, Journal of Allergy and Clinical Immunology: In Practice. (2018) 6, no. 4, 1394–1395, 10.1016/j.jaip.2018.03.005. 29626637

[6] Ariño P. , Aguado L. , Cortada V. , Baltasar M. , and Puig M. M. , Cold Urticaria Associated with Intraoperative Hypotension and Facial Edema, Anesthesiology. (1999) 90, no. 3, 907–909, 10.1097/00000542-199903000-00035. 10078694

[7] Dewachter P. , Kopac P. , Laguna J. J. et al., Anaesthetic Management of Patients With Pre-Existing Allergic Conditions: A Narrative Review, British Journal of Anaesthesia. (2019) 123, no. 1, e65–e81, 10.1016/j.bja.2019.01.020. 30916009

[8] Bizjak M. , Košnik M. , Dinevski D. et al., Risk Factors for Systemic Reactions in Typical Cold Urticaria: Results from the COLD-CE Study, Allergy. (2022) 77, no. 7, 2185–2199, 10.1111/all.15194. 34862605

[9] Handa S. , Dogra S. , and Kumar B. , Comparative Efficacy of Cetirizine and Fexofenadine in the Treatment of Chronic Idiopathic Urticaria, Journal of Dermatological Treatment. (2004) 15, no. 1, 55–57, 10.1080/09546630310013450. 14754652

[10] Bindu B. , Bindra A. , and Rath G. , Temperature Management Under General Anesthesia: Compulsion or Option, Journal of Anaesthesiology Clinical Pharmacology. (2017) 33, no. 3, 306–316, 10.4103/joacp.JOACP_334_16. 29109627 PMC5672515

[11] Agbenyefia P. , Shilliam L. A. , Stoicea N. , Roth A. , and Moran K. R. , Perioperative Management of a Patient with Cold Urticaria, Frontiers of Medicine. (2017) 4, 10.3389/fmed.2017.00222. PMC5741599 29326933

